# Forensic Characteristics of Body Abandonment by Housemates in Japan

**DOI:** 10.7759/cureus.59664

**Published:** 2024-05-05

**Authors:** Haruaki Naito, Yasuhiro Kakiuchi, Yu Kakimoto, Motoki Osawa

**Affiliations:** 1 Department of Forensic Medicine, Tokai University, Isehara, Isehara, JPN; 2 Department of Forensic Medicine, Kindai University, Osakasayama, JPN

**Keywords:** legal autopsy, japan, social withdrawal, hikikomori, abandoned body

## Abstract

Introduction: In Japan, many cases occur wherein housemates fail to report dead bodies found in their homes. However, only individual cases are reported through press and court records, and analysis including unreported cases has not been conducted. In this study, we evaluated cases handled by our Forensic Science Department in which housemates did not immediately report a dead body found in their home. We analyzed the overall picture and forensic characteristics of such cases, stratifying whether the abandoners were estimated hikikomori.

Methods: Of the 1,179 legal autopsy cases handled by the Department of Forensic Medicine of Tokai University from January 1, 2017, to July 1, 2023, we evaluated 45 cases in which housemates did not immediately report dead bodies. The characteristics analyzed were body age, cause of death, autopsy findings, duration from the body’s discovery by the abandoner to the police report, the reason for the lack of report in the first body discovery by the abandoner, and the reason for the report. In this study, the criteria for estimating whether a hikikomori abandoned the body were (1) the police provided the information that the person was a hikikomori or (2) the person met the following four criteria: 20-64 years old, unemployed, not in school, and living with parents.

Results: Positive significant differences were found in the body’s decomposition and the time from the body’s discovery to the report to the police when the abandoner was suspected to be a hikikomori for more than one, four, or eight days. No significant differences were found in the cause of death. Regarding the reported characteristics, when the abandoner was an estimated hikikomori, positive and significant differences were found for recognizing the body and did not report immediately due to shock. Conversely, negative and significant differences were found for the person who reported as the abandoner.

Conclusion: This is the first study that reports on body abandonment by housemates and elaborates on its complications to forensic doctors. The incidence rate of abandonment is higher than expected. This study suggests that hikikomori are more likely to hide the bodies for longer, which hinders the death cause investigation.

## Introduction

Since the term "hikikomori" became famous worldwide, its existence has been reported in other countries, especially in Asia [1-4]. However, while young hikikomori have been the focus of attention, Japan is characterized by a large number of middle-aged hikikomori living with their parents. In Japan, many cases occur where a dead body is found in a house, but the housemate does not immediately report it. This has attracted attention since around 2017 as a typical example of the “80-50 problem,” a problem that occurs in households where older parents care for middle-aged hikikomori [5,6]. In a 2019 Japanese survey, approximately 0.8% of 40-64-year-olds were middle-aged hikikomori living with their parents, indicating that approximately 300,000 households in Japan comprise older parents and middle-aged hikikomori [7]. Although no official data exist on the number of cases of body abandonment by housemates, the nationwide compilations of articles and court records reported that approximately 70 cases occurred over a three-year period [5,6].

In contrast, a large forensic facility reported that approximately 20-30 cases of delayed reporting of bodies by housemates occurred annually in Osaka City alone (approximately 2% of the Japanese population), suggesting that the actual number may be approximately 50 times higher than that reported in the previous study [5,6,8]. This implies that most cases of body abandonment by housemates are not the subject of broadcasts or court procedures. In other words, only forensic doctors dealing with unpublished cases can clarify the epidemiological nature of these cases.

Delayed reporting of a dead body in a household is, by that fact alone, likely to be the subject of investigation by judicial officials and forensic doctors [9]. To what extent and why do highly problematic cases of body abandonment occur in Japan, and are they more common among households with hikikomori? In this study, we evaluated a group of cases handled by the Department of Forensic Medicine of Tokai University in which a housemate did not immediately report a dead body at home and analyzed the characteristics of the bodies and reports.

## Materials and methods

**Figure 1:**
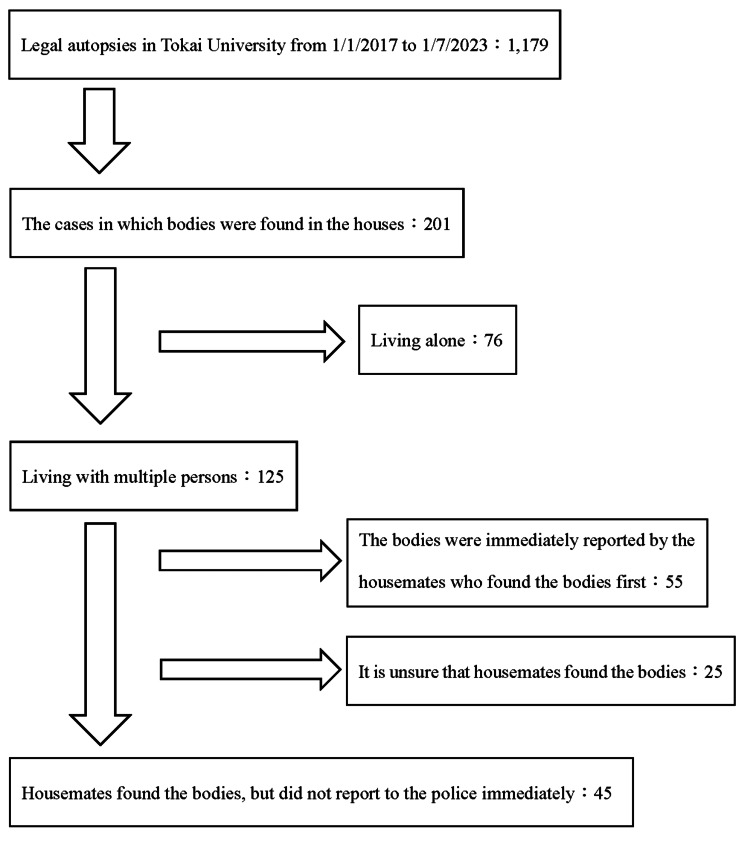
Flowchart of extraction process on body abandonment cases by housemates

A total of 1,179 legal autopsy cases were handled by our department from January 2017 to July 2023. Among these cases, 45 involved body abandonment by housemates. In this study, the housemate who left the body in the home was referred to as the “abandoner.” Figure 1 shows the flowchart of the extraction process on body abandonment cases by housemates. Of the 1,179 legal autopsy cases, the bodies were found in the houses with multiple persons in 125 cases. We excluded 55 cases where the bodies were immediately reported by the housemates who found the bodies first because it was not body abandonment. Additionally, we excluded 25 cases where it was unsure that housemates found the bodies. The cases included the deaths in house fire and the cases where multiple bodies were found in a house, but detailed information about body abandonment could not be collected or estimated by the investigation of the police and forensic doctors. The characteristics analyzed were body age, cause of death, autopsy findings, duration from the body’s discovery by the abandoner to the police report, the reason for the lack of report in the first body discovery by the abandoner, and the reason for the report. These statistics were stratified according to whether the abandoner was an estimated hikikomori. The definition of the estimated hikikomori in this study is discussed below. For the 10 cases involving multiple abandoners not reporting a single body, the body characteristics were counted in duplicate for each abandoner in the abandoner-stratified analysis. The difference in proportions between the case and control groups was analyzed using the chi-square test and Fisher’s exact test. The analysis was conducted by EZR ver1.55, and a p value of ≤0.05 was considered statistically significant [10]. The data of the 45 cases that supported this study are available from the corresponding author upon reasonable request, limiting to the analyzed factors. The ethics committee of Tokai University approved the study protocol and issued the number 23R076-001H.

Definition of estimated hikikomori

One of the major limitations of this study is that the information used to determine whether an abandoner is a hikikomori is ambiguous. The Ministry of Health, Labour and Welfare in Japan lists three definitions of hikikomori: unemployed, not in school, and not going out with communication for more than six months [11]. Japanese and foreign studies of hikikomori, both those that follow the above definitions and those that focus on self-help group participants or psychiatric patients, are mixed [1,12-18]. Although this study focuses on the abandonment of bodies by hikikomori, it is not easy to know the details of the interpersonal relationships of those who abandoned the bodies. Therefore, in this study, fulfilling either (1) or (2) is defined as an estimated hikikomori: (1) information is obtained from the police that the person is a hikikomori or (2) the person meets the following four criteria: 20-64 years old, unemployed, not in school, and living with parents. Neither (1) nor (2) follows the strict definition of a hikikomori. However, if the abandoner or their relatives see them as hikikomori, or if an adult is unemployed and living with their parents, it is likely to indicate some degree of a lack of social interaction. As this study focuses on whether highly problematic body abandonment is observed when the abandoner's sociability is low, we consider that the fulfillment of these conditions is sufficient at this time.

## Results

Table 1 shows the characteristics of the abandoned bodies (age, cause of death, autopsy findings, and duration from the body's discovery by the abandoner to the police report). When the abandoner was an estimated hikikomori, positive significant differences were found for decomposition and more than one day, four days, or eight days from the discovery of the body by the abandoner to the report to the police. No significant differences were found in the cause of death.

**Table 1:** Characteristics of the abandoned bodies The data are presented as the number of participants (n) and percentage (%)

Variables		Total	Abandoners		
			Estimated hikikomori	Others	p value
Number		56 (100%)	20 (100%)	36 (100%)	-
Age (average, median)		38-93 (79, 84)	63-93 (85, 86)	38-91 (76, 80)	-
Cause of death	Natural	22 (39%)	9 (45%)	13 (36%)	0.514
	Homicide (including suspicion)	13 (23%)	2 (10%)	11 (31%)	0.106
	Accident	6 (11%)	2 (10%)	4 (11%)	1.000
	Unknown	15 (27%)	7 (35%)	8 (22%)	0.301
Autopsy findings	Bedsore	13 (23%)	4 (20%)	9 (25%)	0.752
	Bedsore largely related to death	7 (13%)	1 (5%)	6 (17%)	0.239
	Decomposition	27 (48%)	14 (70%)	13 (36%)	0.015
Duration from the body's discovery by the abandoner to the police report	Average	45 days	62 days	34 days	0.329
	<1 day	23 (41%)	2 (10%)	21 (58%)	0.0004
	1-3 days	3 (5%)	1 (5%)	2 (6%)	1.000
	4-7 days	10 (18%)	5 (25%)	5 (14%)	0.468
	8-14 days	7 (13%)	5 (25%)	2 (6%)	0.085
	15-29 days	0 (0%)	0 (0%)	0 (0%)	1.000
	30-89 days	6 (11%)	3 (15%)	3 (8%)	0.655
	≧90 days	7 (13%)	4 (20%)	3 (8%)	0.234
	≧4 days	30 (54%)	17 (85%)	13 (36%)	0.0004
	≧8 days	20 (36%)	12 (60%)	8 (22%)	0.005

Table 2 shows the characteristics of the reports (the reason for the lack of a report in the first body discovery by the abandoner and the reason for the report). When the abandoner was an estimated hikikomori, positive and significant differences were found for recognizing the body and not reporting immediately due to shock, while negative and significant differences were found for the person who reported was the abandoner.

**Table 2:** Characteristics of the reports to the police The data are presented as the number of participants (n) and percentage (%)

Variables			Total	Abandoners		
				Estimated hikikomori	Others	p value
Number			55 (100%)	20 (100%)	35 (100%)	-
The reason for the lack of report in the first body discovery by the abandoner	Recognizing the body	Total	27 (49%)	14 (70%)	13 (37%)	0.039
		Shock	8 (15%)	6 (30%)	2 (6%)	0.021
		Cleaning the room; working and was busy	3 (5%)	0 (0%)	3 (9%)	0.293
		Unknown (dead or unconscious)	8 (15%)	4 (20%)	4 (11%)	0.443
		Unknown (missing)	2 (4%)	2 (10%)	0 (0%)	0.128
		Unknown (did not answer)	6 (11%)	2 (10%)	4 (11%)	1.000
	Could not recognize the body		17 (31%)	4 (20%)	13 (37%)	0.308
	The suspect in the murder		11 (20%)	2 (10%)	9 (26%)	0.293
The reason for the report	Abandoner	Total	15 (27%)	1 (5%)	14 (40%)	0.005
		Recovered from shock	2 (4%)	1 (5%)	1 (3%)	1.000
		Implied the existence of the body to others	5 (9%)	0 (0%)	6 (17%)	0.076
		The abandoner reconsidered that the situation should be reported	5 (9%)	0 (0%)	4 (11%)	0.285
		Completed cleaning; consecutive holidays began	3 (5%)	0 (0%)	3 (9%)	0.293
	Housemate	Found the body and immediately reported	6 (11%)	3 (15%)	3 (9%)	0.657
	Outsider	Total	34 (62%)	16 (80%)	18 (51%)	0.070
		Losing contact and visiting	30 (55%)	14 (70%)	16 (46%)	0.145
		Bad smell	1 (2%)	0 (0%)	1 (3%)	1.000
		Rent was unpaid	0 (0%)	0 (0%)	0 (0%)	1.000
		Full of mails	2 (4%)	1 (5%)	1 (3%)	1.000
		The other causes	1 (2%)	1 (5%)	0 (0%)	0.364

## Discussion

When it comes to the challenges related to body abandonment, two critical factors need to be considered. First, there are no official government records on the number of body abandonments by housemates. We have estimated that there is a big gap between the true number and the reported number based on articles and court records [5,6]. As a primary reason, the police investigate every case of body abandonment, but not every case is the subject of arrest in Japan. It is difficult to estimate which case will be the subject of arrest, broadcast, and court records because it depends on the complex factors. This leads to the second challenge, knowing the duration of the body abandonment. Body abandonment itself has problems, even if it is not the subject of arrest. Long duration of body abandonment impedes the investigation of the cause of death, making it harder for forensic doctors and judicial officials to come to a decision.

Incidence rate

First, we estimated the actual number of cases of body abandonment in Japan. In this study, 45 cases were evaluated over a period of six years and six months. Since the annual number of legal autopsies in our department accounted for 1.5% of the total number of legal autopsies in Japan, it was estimated by simple calculations that approximately 460 cases occur annually in Japan as a whole [19,20]. Even after excluding homicide cases (including suspected homicide), 34 cases were included in this study, resulting in an estimated annual incidence of approximately 350 cases. In the Introduction section, we reported that there were approximately 70 cases of body abandonment by housemates over a three-year period, or approximately 23 cases annually, according to articles and court records in Japan [5,6]. In other words, whether we exclude homicide cases or not from our samples, the publicly reported number of cases of body abandonment by housemates may have been underestimated by <10%; this may be because most of these cases are not the subject of broadcasts or court procedures, even though such cases may be suspected of criminality and more likely to be the subject of police investigations and legal autopsies. In this study, it was estimated that approximately 470 cases occur annually in Japan, and approximately 40% of the deaths are natural deaths. Therefore, numerous body abandonment cases may not have been the subject of police investigations and legal autopsies if reported at the time of body discovery.

Duration from the discovery of the body by the abandoner to the report to the police

As shown in Table 1, the biggest problem for the estimated hikikomori group was longer body abandonment times. In most of the body abandonments by estimated hikikomori, the duration from the discovery of the body by the abandoner to the report to the police was greater than or equal to four days, while most of those by the control group were within one day. In addition, 70% of the bodies in the estimated hikikomori group were found to be decomposed, reflecting a long duration of body abandonment. The difference in unknown deaths was nonsignificant, but this is because some of the decomposed cases were determined not to have been unknown deaths. If more samples are collected, a significant difference may be found in the case of unknown deaths, reflecting the long body abandonment times, and we can assume that it impedes the investigation of the cause of death.

Why does the estimated hikikomori leave the bodies unreported for a very long time? As shown in Table 2, in the control group, 40% of the cases were reported by abandoners; in contrast, in the estimated hikikomori group, abandoners reported only 5% of cases. This is an interesting point to consider. The estimated hikikomori group had significantly more cases where the abandoner recognized the body. They tended to passively wait for a visit from an outsider, resulting in long-term body abandonment. Motives such as shock, anxiety about reporting, and unwillingness to act seem to play a significant role in the tendency of hikikomori not to report a dead body even though they recognized it.

There is a mix of “secondary hikikomori” who have comorbid mental symptoms and “primary hikikomori” who do not, and both are possibly important when analyzing cases of body abandonment [21-23]. Secondary hikikomori are associated with high rates of major depression, social anxiety, and avoidant personality disorder [13,22,24-26]. The choice not to report the body after finding it is risky in this society and is unlikely to be seen in patients with mild or moderate mental symptoms. Middle-aged hikikomori with severe and long-term mental symptoms may find it challenging to communicate with the police or receive emergency help. In particular, if they fail to call the police or receive emergency help when they discover the dead body for the first time, they may find it even more difficult to be questioned by the police about the reasons for not reporting and about their own condition.

Primary hikikomori, accounting for 25%-40% of all hikikomori, have also been involved in body abandonment [21,25]. Primary hikikomori is a group of people who avoid social communication but do not meet existing mental illness classifications, such as mood or anxiety disorders [21-23]. Likely, the main reason for primary hikikomori who do not report is not shock or anxiety about reporting but just not wanting to act or finding reporting just bothersome. It is speculated that if the process of struggling with shock and anxiety after the discovery of a body itself is rare among primary hikikomori, it may be common among hikikomori related to long-term body abandonment. This could be one reason for the fact that only 5% of the hikikomori recovered from the shock and voluntarily reported. However, it is not easy to differentiate between primary hikikomori and secondary hikikomori. As shown in Table 2, most reasons for body abandonment were unclear, as 30% of the total number of abandoners and 40% of those in the estimated hikikomori group were dead, unconscious, missing, or left questions unanswered.

Limitations of the study

This study had several limitations. First, although the stratification of hikikomori was based on police information, detailed information could not be obtained in all cases because information on the abandoner could only be obtained from himself, relatives, or neighbors. However, the abandoner was dead, missing, or socially isolated in some cases. In many cases in this study, social information about abandoners was unclear. Therefore, it is likely that many abandoners assigned to the control group would also be assigned to the hikikomori group. As a result, the evidence that estimated hikikomori tend to leave the body unreported might be strengthened, but it is unclear how the trend regarding the number of days of body abandonment might change.

Second, the overall sample size was small, probably obscuring the problematic nature of the number of body abandonment days. The contact and visitation schedules of relatives and nurses often influenced the number of days. For example, it was found that hikikomori tend to leave bodies longer, but whether the nature of hikikomori is truly related to this finding remains unclear. Further studies with larger sample sizes are necessary to confirm this finding.

## Conclusions

In this study, 45 cases of body abandonment by housemates were collected over a period of six years and six months in Tokai University, indicating the possibility that approximately 470 cases of body abandonment by housemates occur annually in Japan. In collected cases, approximately 40% of the deaths were due to natural deaths. When the abandoner was an estimated hikikomori, approximately 90% of the bodies were left for longer than four days, and there were significantly more cases of decomposition because the estimated hikikomori tended to wait passively for a visit by an outsider without voluntarily reporting the body, even though they recognized it.
